# Prevalence, awareness, treatment and control of high blood pressure in a cohort in Northern Andean Peru

**DOI:** 10.1080/16549716.2023.2285100

**Published:** 2023-12-01

**Authors:** Giuliana Sanchez-Samaniego, Stella Maria Hartinger, Daniel Mäusezahl, Jan Hattendorf, Günther Fink, Nicole Probst-Hensch

**Affiliations:** a Department of Epidemiology and Public Health, Swiss Tropical and Public Health Institute, Allschwil, Switzerland; b Faculty of Science, University of Basel, Basel, Switzerland; c School of Public Health and Administration, Universidad Peruana Cayetano Heredia, UPCH, Lima, Peru

**Keywords:** Hypertension, awareness, control, high-altitude, Andes

## Abstract

**Background:**

Gaps exist along the high blood pressure (HBP) diagnosis-treatment-control pathway in high, low and middle-income countries.

**Objective:**

To determine the prevalence of HBP and to describe the levels of awareness, control and treatment of HBP in the rural Peruvian Andes.

**Methods:**

This cross-sectional study is embedded into a multigenerational cohort. We analysed data of all adult participants aged ≥ 30 years (*n* = 2752) who answered a baseline health and lifestyle questionnaire and underwent a physical examination, which included three blood pressure readings. HBP was defined as measured systolic or diastolic blood pressure (BP) ≥140 and/or 90 mm Hg and/or self-reported physician-diagnosed hypertension and/or self-reported antihypertensive intake. The determinants of the prevalence of HBP, unawareness of HBP and uncontrolled HBP were assessed using mixed-effect logistic regressions.

**Results:**

HBP was present in 18.9% of the participants. Of those with measured HBP, 72.2% were unaware of their HBP. Among those with a diagnosed or medically treated hypertension, 58.4% had uncontrolled HBP. The prevalence of HBP was higher in women (OR: 1.12, CI: 1.02–1.24), increased with age (OR: 1.01, CI: 1.01–1.01) and the presence of family history of hypertension (OR: 1.15, CI: 1.08–1.24), and decreased with healthier lifestyle score (OR: 0.93, CI: 0.91–0.95). Unawareness of HBP was lower among women (OR: 0.56, CI: 0.38–0.83), higher among participants living over 3000 m Above Sea Level (OR: 1.15, CI: 1.03–1.27) and decreased with age (OR: 0.99, CI: 0.98–0.99).

**Conclusions:**

Unawareness of HBP was high, few HTN patients received treatment and BP remained high in the presence of antihypertensive treatment.

## Introduction

High blood pressure (HBP) is the leading risk factor for cardiovascular diseases and deaths worldwide [1,2]. HBP is a modifiable risk factor. Its prevention, early detection, and control is critical for preventing adverse cardiovascular outcomes [1]. The prevalence of long-term HBP or hypertension (HTN) decreased considerably from 2000 to 2015 in high-income regions, and improvements were also observed in Central and Eastern Europe, Latin America and the Caribbean [2]; however, unawareness of high blood pressure and uncontrolled blood pressure remain big challenges for health systems across the world [3].

A multinational study conducted in high-, middle- and low-income countries showed that only half of people with HTN were aware of their elevated blood pressure [3]. Another study from the Latin American region using data from population-based cohorts described prevalences of HTN awareness of between 56% and 70% depending on the country studied [4]. Furthermore, a pooled analysis from the Non-Communicable Diseases Risk Factor Collaboration stated that in 2019 less than half of patients receiving antihypertensive treatment globally had controlled blood pressure levels (systolic blood pressure ≤ 140 and diastolic blood pressure ≤90 mm Hg), with a lower proportion of men (18%) achieving controlled blood pressure levels compared to women (23%) [5]. Gaps exist along the diagnosis-treatment-control pathway regardless of the socio-economic level of countries and health care access [3]. Community-based and country-specific information are needed to inform national guidelines for HTN prevention and control [3,4,6].

In Peru, a meta-analysis considering national and subnational studies based on a mix of population-based and not population-based designs estimated a pooled HTN prevalence of 22% [7]. Information regarding awareness and control of HTN at population level is available from two population-based cohort studies (the CRONICAS and PERU MIGRANT cohorts) [8,9] and the National Demographic Health Survey (DHS), but information about people’s health seeking behaviour after knowing their HBP status is unknown [10,11]. In 2018, Peru joined the HEARTS [12,13] in the Americas initiative of the Pan American Health Organization and began following international standards for prevention and control of HBP. This initiative proposed a new model where the management of HBP must occur at the primary-care level. The initiative focuses on healthy lifestyle counselling, evidence-based treatment protocols, access to essential medicines and technology, cardiovascular disease risk-based management, team-based care, and monitoring systems [12,13]. The implementation of this initiative has been piloted in 32 primary care establishments in five regions of Peru [14]. Context-specific data is now needed for supporting the implementation of this initiative and for evaluating its future impact.

This cross-sectional study embedded in the ALTO cohort aimed to determine the prevalence of HBP, HBP unawareness and uncontrolled HBP in adults living in a high altitude rural setting in the Andes. We additionally explored the association of these endpoints with socioeconomic and lifestyle determinants.

## Methods

### Study design and participants

This is a cross-sectional study analysing baseline data provided by adult participants of the Peruvian Andes Multigenerational High Altitude Cohort (ALTO). ALTO was established in the high altitude rural province of San Marcos in northern Andean Peru [15]. San Marcos’ province is located between 1900 and 3900 m above sea level (MASL). Most families live in houses with clay-sand walls and earthen floors. The principal source of income includes farming and livestock trade in rural areas; in the peri-urban areas construction, mining and governmental positions are also important sources of income [16].

The ALTO cohort is built up on index pregnancies. The cohort aimed at recruiting all women living in the San Marcos province with an expected due date or actual delivery between February 2020 and August 2022, as well as their partners and kin (parents and grandparents). Pregnant women as index participants were identified through the health centre’s pregnancy surveillance system and in communities through household visits. Index women who were missed during their pregnancy were enrolled at post-partum. Once enrolled, we filled out the women’s genealogy form that helped to identify their kin. The ALTO cohort enrolled 1994 women (1039 during pregnancy and 955 at post-partum) and 3023 members of their kin representing about 35% of index women’s families. For this study, we included data obtained from all adult participants (post-partum index cases; partners; parents; grandparents) of the cohort who were 30 years of age or older. We excluded pregnant women and women less than 12 weeks post-partum.

All the participants underwent a physical examination and answered questionnaires regarding their socioeconomic information, health and lifestyle characteristics. Data were collected at the participant’s house. Data collection (interviews; health examinations) took place from February 2020 to December 2022. Socioeconomic and household characteristics, lifestyle data, data on personal and family medical history, blood pressure (BP) and anthropometrics measurements were collected following the ALTO protocol [15]. Further details on the BP measurements are provided below.

The fieldwork was conducted by local staff, from the long-term epidemiological research station in San Marcos, with good knowledge of the local culture. These highly experienced fieldworkers were trained and certified in human subject research ethics, in participant’s recruitment and enrolment procedures and in data collection activities including interviewer-administered questionnaires and physical examinations. Fieldworker training lasted 6 weeks. After data collection began, weekly meetings were held with the field coordinator and researchers to address any issues that might have arisen from field data collection. This also helped reinforce data collection procedures.

We obtained ethical clearance from the ethical review board of the Universidad Peruana Cayetano Heredia (N° 192-08-16) and from the Swiss ethics commission, Ethikkommission Nordwest und Zentralschweiz (EKNZ) (Req-2020–00088). All participants gave their written informed consent.

### Questionnaire information

In the questionnaire-based in-person interviews, we collected information on household material, food insecurity (household food insecurity scale) [17], household members’ participation in the Peruvian national cash transfer programmes (JUNTOS, pension 65, both or none [18]) and socioeconomic status using the unsatisfied basic needs score [19]. Additionally, we collected the altitude of residence (<2500, 2500–3000, >3000 MASL).

Lifestyle data included information on smoking (does not smoke, occasionally, daily, weekly), fruit and vegetable intake (≥ vs. <5 portions per day), alcohol consumption (harmful drinking (yes/no)) [20], and physical activity (low, moderate, vigorous activity) [21]. Data on smoking, fruit and vegetable intake were collected using the WHO STEPS Instrument [22]. Alcohol consumption among those reporting ever consuming alcohol was measured using the Alcohol Use Disorder Identification Test score. Participants with a score ≥ 8 were classified as having ‘harmful drinking’ [20]. The physical activity was measured and classified using the International Physical Activity Questionnaire [21].

We collected information on personal health morbidity by asking: ‘Has a doctor ever told you that you have one of the following diseases?’, followed by a list of diseases, including most importantly HTN, but also type-II diabetes, high cholesterol, stroke and heart disease. Participants who reported having a physician-diagnosed HTN were asked about their medication intake, which was classified as: no medicine, antihypertensive medicine (yes/no), other medicines (i.e. statins and aspirin) (yes/no) and traditional treatment (yes/no). We additionally obtained information on antihypertensive intake by asking patients with self-reported heart disease and/or stroke about their medication and by asking all participants to list any medications that they had taken in the previous 3 months. We used the list of medications available in the local health centre to help participants identify their medicine. Antihypertensive drugs were considered and classified as angiotensin-converting-enzyme (ACE) inhibitors, angiotensin-II receptor blockers (ARBs), beta-blockers (BBs), calcium antagonists, alpha-2 adrenergic agonist (AAAs) and diuretics. Additionally, we collected information on the family health history of HTN, type-II diabetes, stroke and heart disease. Daughters, sons, parents, siblings, grandparents and biological aunts and uncles were considered as family.

### Clinical measurements

We used standardised techniques to measure BP and anthropometrics [22]. Trained staff measured BP on the left arm (supported at heart level) using an automatic BP meter (OMRON HEM-712C). Before the measurement, participants reposed for at least 5 min sitting down and were instructed not to talk or cross their legs. To reduce the ‘white coat’ effect, BP measurements were taken three times with a one-minute interval between measurements; the average of the last two measurements was used for analysis [23].

Weight and height were measured using a calibrated SECA 802 scale and a portable SECA stadiometer. Devices were placed on a hard and flat surface to ensure a levelled horizontal position. Participants were asked to remove their footwear, headgear (hats, caps, and ribbons), high hairdos and to take off their belts, and empty their pockets. In order to measure weight, participants stepped onto the weight scale with one foot on each side of the scale. They were asked to stand still, face forward, place their arms on their side and wait until the fieldworker registered the reading. For height measurments, participants stood on the stadiometer board with their feet together, heels against the backboard and knees straight, looking straight ahead without tilting their head. The fieldworker then moved the headpiece down to touch the crown of the participant’s head and read the height in centimetres at the exact point to the nearest millimetre. We repeated the weight and height measurements twice to maximise data reliability. The average of the two measurements was used for analysis. Body mass index (BMI) was calculated as weight (kg)/height (m)^2^. Participants were classified as underweight (<18.5 kg/m^2^), normal weight (18.5 to <25 kg/m^2^, overweight (25 to <30 kg/m^2^) and obese (≥30 kg/m^2^).

## Data analysis

### Primary outcomes - related to HBP prevalence and HTN control

HBP was defined as having systolic BP ≥140 mmHg and/or diastolic BP ≥ 90 mmHg (BP ≥ 140/90 mmHg) in the physical examination; and/or having a physician-diagnosed HTN; and/or self-reported antihypertensive intake. We measured BP three times during one visit. Yet a formal HTN diagnosis requires BP measurements from at least two separate occasions [24]. We therefore refer to our primary outcome as HBP rather than HTN.

To evaluate the local HTN control efficiency from screening to diagnosis and treatment, the following secondary HTN endpoints were defined:

- **HBP unawareness**: participants who did not self-report a physician’s diagnosed HTN and/or antihypertensive medication intake among participants with HBP measured in the physical examination of this study (BP ≥ 140/90 mmHg).
- **Physician-diagnosed HTN**: participants who answered ‘yes’ to the question ‘Has a doctor ever told you that you have hypertension?’
- **Antihypertensive intake**: participants who self-reported intake of any antihypertensive medication
- **Controlled HBP**: participants with BP measurements < 140/90 mm Hg in this study among participants with a physician-diagnosed HTN and/or antihypertensive intake.
- **Uncontrolled HBP**: participants with BP measurements ≥ 140/90 mm Hg in this study among participants with a physician-diagnosed HTN and/or antihypertensive intake.

### Healthy lifestyle score (LS)

In order to examine compliance with the national clinical guidelines for HTN prevention [24], we collected information on participants’ lifestyle characteristics in order to create a lifestyle score for inclusion in the models. We used the following variables to create the score: not smoking daily or weekly; non-harmful alcohol consumption; consumption of at least five portions of fruits and vegetables per day; moderate or high physical activity; and BMI <25 kg/m^2^. We assigned one point for each healthy lifestyle behaviour and used the sum score for analysis (range 0–5).

### Statistical analysis

Results are reported as numbers or percentages for categorical variables and median (interquartile range) for continuous variables. Given the small number of missing data (4%), complete case analysis was applied rather than imputing missing covariate data.

The LS, as a measure of primary prevention, was calculated for: a) all participants, and b) the subgroup without HBP (measured BP < 140/90 mm Hg) and without physician diagnosed-HTN and without antihypertensive intake.

Prevalences for primary outcomes HBP, HBP unawareness and uncontrolled HBP were calculated.

Three separate mixed-effect logistic regressions were applied to the data for assessing the determinants of primary outcomes HBP, HBP unawareness and uncontrolled HBP. Participant’s household was included in the model as random-effect.

Prevalence calculations and the logistic regressions were based on different participant samples. First, for prevalence and determinants of HBP all participants were included. Second, for prevalence and determinants of HBP unawareness participants with BP ≥ 140/90 mm Hg during the physical examination were included. Third, for prevalence and determinants of uncontrolled HBP, participants with a physician-diagnosed HTN and/or antihypertensive intake were included. In all models participant’s household was considered a random effect to account for potential correlation within households. According to previous literature, we a priori included the following variables of interest as fixed-effects in the models: sex (male, female); age (years as continuous variable); family history of HTN (yes, no); healthy lifestyle score [25] (as continuous score); and in line with previous research in Peru [8,11] the two location-specific covariates participation in a cash transfer programme (yes, no) and altitude of living (<2500; 2500–3000; >3000 MASL; categorical). In building the respective models, we tested each of these predictors separately for interaction with sex. We observed statistically significant interactions between sex and age (p-value_interaction_ <0.05) in the models for HBP and HBP unawareness and according interaction terms were therefore included in these two models.

In a separate analysis, additional covariates including self-reported diagnosis of high cholesterol, diabetes and HBP treatment (none, antihypertensive treatment; other treatment) were added to the adjusted models. HBP treatment was only added to the uncontrolled HBP model. Using the Akaike Information Criterion, we observed that adjusting the models for these additional covariates did not improve the fit of any of the three models, and these variables were therefore not included in the models reported.

Lastly, we conducted a sensitivity analysis for the model on HBP to account for potential overestimation of HBP prevalence when including self-reported physician-diagnosed HTN in the definition of HBP. This additional analysis excluded participants who only self-reported physician-diagnosed HTN, but did not report any intake of antihypertensive intake and did not have measured BP ≥ 140/90 mmHg (*n* = 61).

Analyses were performed using R core team version 4.1.3. (2022). All p-values <0.05 were considered statistically significant.

## Results

### Characterisation of the study population

**Figure 1.**
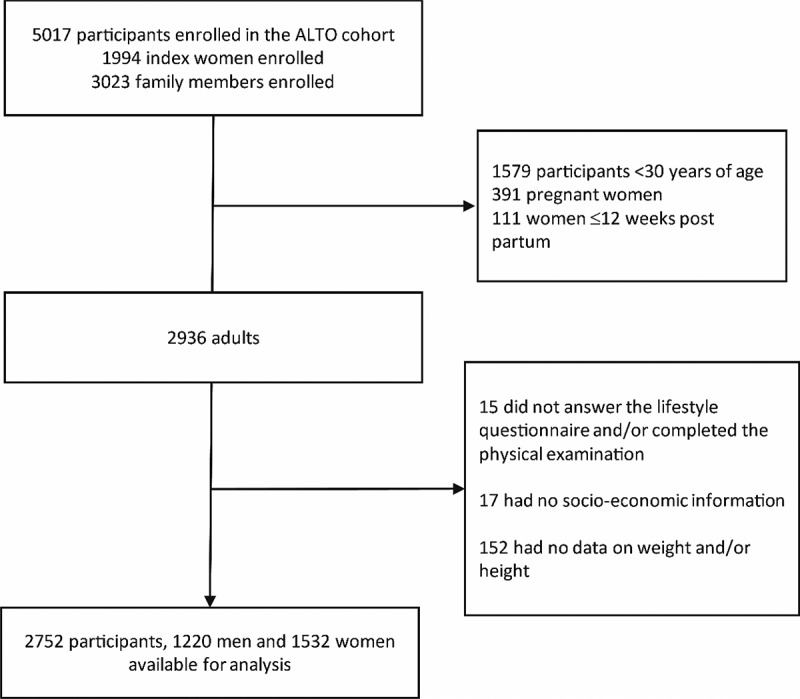
Flow diagram for the study participants as part of the Peruvian Andes multigenerational high altitude cohort (ALTO).

A total of 5017 subjects were enrolled in the ALTO cohort, 1579 were <30 years of age, and 391 were pregnant women and 111 women were <12 weeks post-partum. Of the 2936 subjects meeting the inclusion criteria, 15 did not answer the lifestyle questionnaire and/or completed the physical examination, 17 did not have socioeconomic information and 152 had incomplete weight and height measurements. The study analysed data of 2752 participants (Figure 1).

Of the 2752 participants, 1532 (55.7%) were women and 1220 (44.3%) were men. The median age of the study population was 53 years with age range from 30 to 97 years. Table 1 describes the distribution of demographic characteristics, lifestyle, comorbidities, family health history and household characteristics among the study participants overall and separately for each sex. Of all participants, 89.0% had a LS of 3 or 4 (women: 92.0%; men: 86.7%) (Table 1). The distribution of LS in the absence of HBP was similar, with 90.2% participants in the 3 or 4 score groups (women: 92.7%; men: 87.1%) (data not shown).

**Table 1.** Characteristics of study participants.

	Women	Men	All participants
N (%)	1532 (55.7)	1220 (44.3)	
Age (years)	53 (42, 65)	53 (40, 66)	53 (41, 66)
Systolic BP (mm Hg)	114 (104,126)	118 (108, 128)	115 (106,128)
Diastolic BP (mm Hg)	74 (67, 82)	75 (68, 82)	74 (68, 82)
BP ≥140/90 during physical examination	239 (15.6)	193 (15.8)	432 (15.7)
Physician-diagnosed HTN	135 (8.8)	72 (5.9)	207 (7.5)
Antihypertensive intake	31 (2.5)	43 (2.8)	74 (2.7)
High blood pressure*	298 (19.5)	223 (18.3)	521 (18.9)
**Lifestyle characteristics**			
Harmful drinking	0	48 (3.9)	48 (1.7)
Smoking			
Does not smoke	1529 (99.8)	1087 (89.0)	2616 (95.0)
Smokes daily	0	16 (1.3)	16 (0.6)
Smokes weekly	2 (0.1)	102 (8.4)	104 (3.8)
Occasional smoker	1 (<0.1)	15 (1.2)	16 (0.6)
Physical activity			
Low	233 (15.2)	140 (11.5)	373 (13.6)
Moderate	533 (34.8)	204 (16.7)	737 (26.8)
High	766 (50.0)	875 (71.8)	1641 (59.7)
Fruit and vegetable consumption (≥5 portions per day)	43 (2.8)	36 (3.0)	79 (2.9)
BMI (kg/m^2^)			
<18.5	14 (0.9)	13 (1.1)	28 (1.0)
18.5–24.9	558 (36.4)	626 (51.4)	1184 (43.0)
25–29.9	641 (41.6)	484 (39.7)	1125 (40.9)
≥30	319 (20.8)	96 (7.9)	415 (15.1)
Lifestyle score			
1–2	121 (7.9)	148 (12.2)	269 (8.9)
3	927 (61.0)	558 (45.7)	1485 (54.0)
4	468 (31.0)	500 (41.0)	968 (35.0)
5	16 (1.1)	14 (1.1)	30 (1.1)
**Comorbidities (self-reported)**			
Diabetes	23 (1.5)	8 (0.7)	31 (1.1)
Heart disease	17 (1.1)	17 (1.4)	34 (1.2)
Stroke	8 (0.5)	12 (1.0)	20 (0.7)
High cholesterol	48 (3.1)	18 (1.5)	66 (2.4)
**Family health history (self-reported)**			
HTN family history	68 (4.4)	45 (3.7)	113 (4.1)
Diabetes family history	38 (2.3)	28 (2.3)	66 (2.4)
Stroke family history	41 (2.7)	37 (3.0)	78 (2.8)
Heart disease family history	44 (2.9)	23 (1.9)	67 (2.4)
**Household characteristics**			
Access to sewage system	161 (10.5)	112 (9.2)	273 (9.9)
Earthen Floor	1254 (81.9)	975 (80.0)	2229 (81.0)
Roof with tiles	1107 (72.3)	869 (71.3)	1976 (71.8)
Biomass fuel use	1351 (88.2)	1036 (85.0)	2387 (86.8)
Household food security			
Food secure	1266 (82.6)	1043 (85.6)	2309 (83.9)
Mildly food insecure	194 (12.7)	139 (11.4)	333 (12.1)
Moderately food insecure	15 (1.0)	7 (0.6)	22 (0.8)
Severely food insecure	57 (3.7)	30 (2.5)	87 (3.2)
Government aid			
No aid	919 (60.0)	748 (61.4)	1667 (60.6)
Pension 65 (P65)	261 (17.0)	188 (15.4)	449 (16.3)
JUNTOS	335 (22.9)	270 (22.1)	605 (22.0)
P65 and JUNTOS	17 (1.1)	13 (1.1)	30 (1.1)
Unsatisfied basic needs			
0	1158 (75.6)	931 (76.4)	2089 (75.9)
1	345 (22.5)	264 (21.7)	609 (22.1)
2	27 (1.8)	22 (1.8)	49 (1.8)
3	2 (0.1)	2 (0.2)	4 (0.1)
Altitude of living (MASL)			
<2500	442 (28.9)	374 (30.7)	816 (29.7)
2500–3000	465 (30.4)	381 (31.3)	846 (30.8)
>3000	625 (40.8)	464 (38.1)	1089 (39.6)

Data presented as *n* (%), or mean (interquartile range). BP, blood pressure; HTN, hypertension; BMI, body mass index; JUNTOS, National cash transfer programme; P65, Pension 65 cash transfer programme; MASL, metres above sea level.

*High blood pressure defined as having systolic BP ≥ 140 mm Hg and/or diastolic BP ≥ 90 mm Hg in the physical examination; and/or having a physician-diagnosed HTN; and/or self-reported antihypertensive intake. *High blood pressure defined as having systolic BP ≥ 140 mm Hg and/or diastolic BP ≥ 90 mm Hg in the physical examination; and/or having a physician-diagnosed HTN; and/or self-reported antihypertensive intake. *High blood pressure defined as having systolic BP ≥ 140 mm Hg and/or diastolic BP ≥ 90 mm Hg in the physical examination; and/or having a physician-diagnosed HTN; and/or self-reported antihypertensive intake.

### Prevalence of HBP and its subgroups

**Figure 2.**
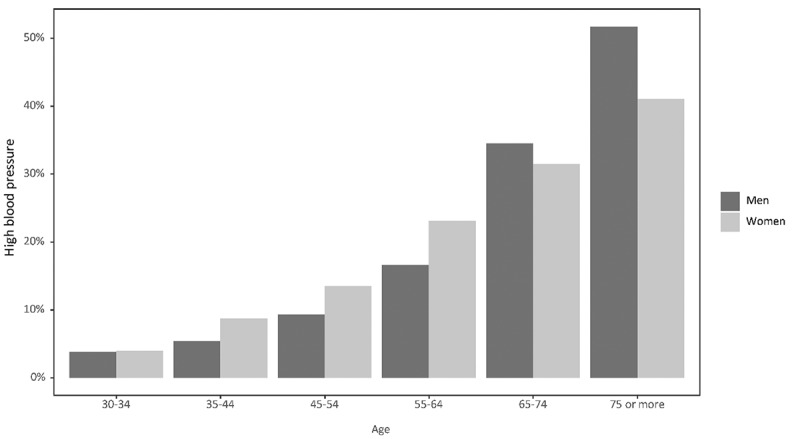
Distribution of the prevalence of high blood pressure (measured blood pressure ≥ 140/90 mmHg or self-reported physician-diagnosed hypertension or self-reported intake of antihypertensive medication), by sex and age category.

As shown in Table 1, we found that 15.7% (432/2752) of participants had a BP ≥ 140/90 mmHg during the physical examination, with 7.5% (207/2752) of all participants reporting a physician-diagnosed HTN. Similar prevalences of BP ≥ 140/90 mmHg based on the physical examination were observed among women (15.6% (239/1532)) and men (15.8% (193/1229)). In contrast, women showed a slightly higher prevalence of physician-diagnosed HTN than men (8.9% (135/1752) vs. 5.9% (72/1752)). HBP defined as a combination of the presence of measured HBP and/or self-reported physician-diagnosed HTN and/or intake of antihypertensive medication had a prevalence of 18.9% (521/2752) (women: 19.5% (298/1532); men: 18.3% (223/1220)) and increased steeply with age, reaching a prevalence of over 50% in men ≥75 years and of over 40% in women ≥75 years (Figure 2).

**Figure 3.**
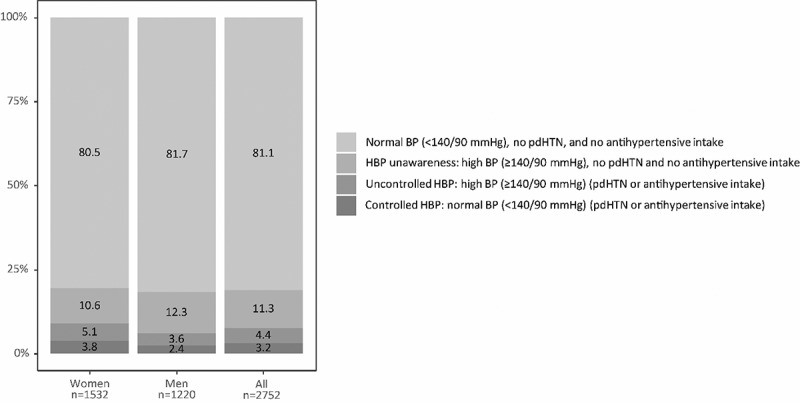
Unaware, uncontrolled, controlled and healthy blood pressure participants among study participants, overall and stratified by sex (%) (pdHTN: physician-diagnosed HTN, HBP: high blood pressure, BP: blood pressure).

Figure 3 shows the number of participants in the HBP groups: participants with no BP ≥ 140/90 mm Hg, no physician-diagnosed HTN, and no antihypertensive intake; participants unaware of HBP; participants with uncontrolled HBP; participants with controlled HPB. In the total study population, 11.3% were unaware of HBP, 4.4% had uncontrolled HBP, and 3.2% had their HBP controlled.

**Figure 4.**
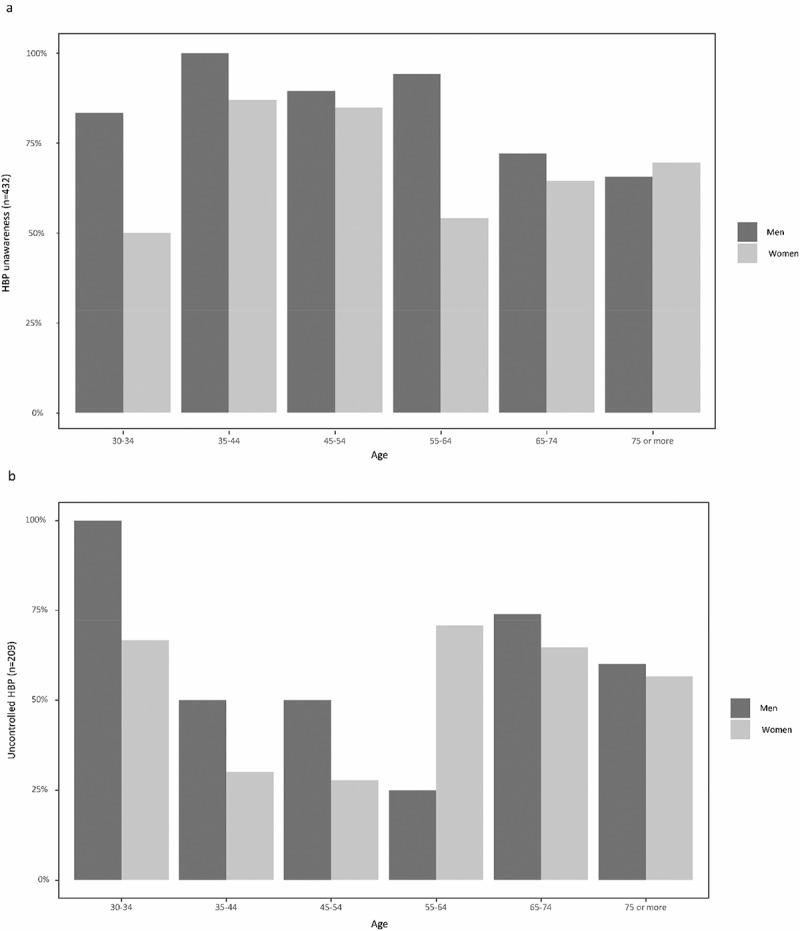
Prevalence of unawareness of high blood pressure (a) and uncontrolled high blood pressure (b) among women and men by age (%) (HBP: high blood pressure).

Among subjects with measured high BP ≥ 140/90 mm Hg, 72.2% (312/432) were unaware of their condition (Table 1 and Figure 3). Men presented a higher frequency of unawareness than women (77.7% (150/193) vs. 67.8% (162/239)). Of those with a physician-diagnosed HTN or intake of antihypertensive medication, 58.4% (122/209) were uncontrolled. The prevalence of uncontrolled HBP was higher in men (60.3% (44/73)) than in women (57.4% (78/136)). Figure 4 displays the sex and age-specific prevalences of unawareness of HBP (a) and uncontrolled HBP (b). Unawareness of HBP was higher in men in almost all age categories. A suggestion of a reduction in the prevalence of unawareness of HBP was observed from the age of 55 onwards in women and from the age 65 onwards for men (Figure 4(a)). Uncontrolled HBP prevalence was higher in men in all age groups except 55–64 olds (Figure 4(b)). The prevalence of uncontrolled blood pressure increased from age 55 onwards in women, and from age 65 onwards in men.

### Use of antihypertensive medicine in patients self-reporting a physician-diagnosed HTN

Only 34.8% (72/207) of the participants with a physician-diagnosed HTN were taking antihypertensive medications (irrespective of other medicines); 10.1% (21/207) received other treatment and 1.9% (4/207) took only other medicines (i.e. statins and aspirin); 8.2% (17/207) took only traditional medicines; 55.1% (114/207) were not taking any medicines. The majority of participants were taking ACE inhibitors (77.8% (56/72)), 12.5% (9/72) were taking only ARBs, 5.6% (4/72) only BBs and 1.4% (1/72) took only diuretics. Only two participants (2.8%) were taking BBs and diuretics combined. All the medications identified were available at the local public health centre and were included in their basic list of drugs.

**Figure 5.**
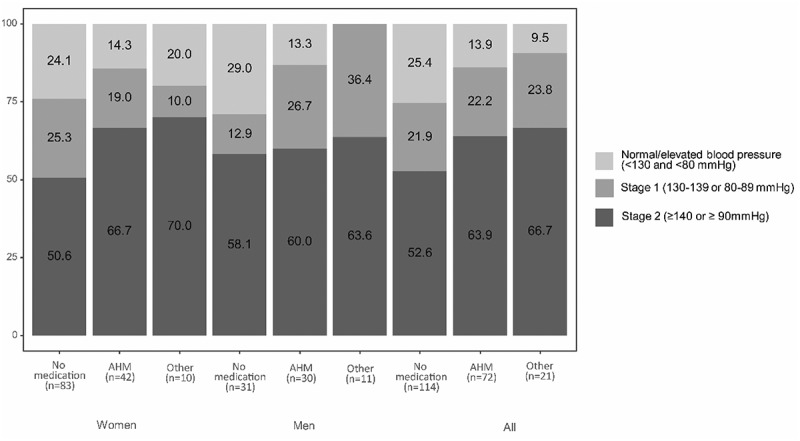
Blood pressure levels according to type of treatment among participants with a physician-diagnosed hypertension. (AHM: Antihypertensive medication intake with or without other medication, other medication: Statins, aspirins and traditional medication).

Among the persons treated with antihypertensive medication, irrespective of sex, the majority had Stage 1 (systolic BP: 130–39 mmHg or diastolic BP: 80–89 mmHg) or Stage 2 HTN (≥140/90 mmHg) (Figure 5). Among persons not self-reporting any medication more than half exhibited BP levels indicative of at least stage 2 HTN. Two thirds of participants taking other medication (i.e. statins, aspirins and traditional medication) had Stage 2 HTN. All men taking other medication had at least stage 1 HTN; in women taking other medication, 80% qualified as stage 1 HTN. The highest percentages of participants with normal to only elevated BP (systolic BP ≤ 130 mmHg and diastolic BP ≤ 80 mmHg) were observed in the group without self-reported medication.

### Determinants of HBP, unawareness of HBP, and uncontrolled HBP

Table 2 presents results from the three mixed-effect logistic regressions for the three primary endpoints evaluated in different subgroups. The three models are controlled for age, sex, LS, family history of HTN, government aid, and altitude as fixed-effects and participant’s household as random-effect. Associations did not materially change when excluding participants with only a physician-diagnosed HTN (*n* = 61) from the model on HBP.

**Table 2.** Factors independently associated with high blood pressure, unawareness of high blood pressure and uncontrolled high blood pressure.

	HBP (*n *= 2752)	HBP unawareness (*n *= 432)	Uncontrolled HBP (*n *= 209)
	OR (CI)	OR (CI)	OR (CI)
Age (years)	1.01 (1.01–1.01)	0.99 (0.98–0.99)	1.03 (1.00–1.01)
Sex (Women)	1.12 (1.02–1.24)	0.56 (0.38–0.83)	1.02 (0.87–1.17)
Age*sex	0.99 (0.99–0.99)	1.01 (1.01–1.02)	
Lifestyle score	0.93 (0.91–0.95)	1.06 (1.00–1.14)	0.90 (0.93–1.13)
Family history of HTN	1.16 (1.08–1.24)	0.82 (0.67–1.00)	0.32 (0.66–1.03)
Government aid	0.99 (0.96–1.02)	1.01 (0.93–1.11)	1.31 (0.96–1.29)
Altitude of living			
<2500 MASL			
2500–3000 MASL	1.01 (0.97–1.05)	1.04 (0.93–1.15)	1.42 (0.88–1.20)
>3000 MASL	1.00 (0.97–1.04)	1.15 (1.03–1.27)	1.15 (0.86–1.21)

HBP, High blood pressure; HTN: hypertension; SE: Standard error; OR, Odds Ratio; CI, Confidence Intervals; MASL, metres above sea level.

The odds of HBP increased with age (OR: 1.01, CI: 1.01–1.01) and the odds of HBP was higher for women than for men (OR: 1.12, CI: 1.02–1.24) (Table 2). Additionally, the interaction term indicated that men’s odds of HBP surpassed those of women’s after the age of 55 (Supplementary information 1). A family history of HTN increased the odds of HBP prevalence (OR: 1.16, CI: 1.08–1.24) whereas the odds decreased with higher LS (OR: 0.93, CI: 0.91–0.95). Furthermore, living in a household receiving governmental aid (OR: 0.99, CI: 0.96–1.02) and the altitude of living (OR: 1.01, CI: 0.97–1.05 and OR: 1.00, CI: 0.97–1.04) were not statistically significantly associated with the prevalence of HBP.

In the model analysing the prevalence of being unaware of ones HBP, we observed that women (OR: 0.56, CI: 0.38–0.83) were less likely to be unaware of their HBP. Furthermore, the odds of being unaware of HBP decreased with age (OR: 0.99, CI: 0.98–0.99). The interaction between age and sex indicated that the sex difference in HBP unawareness decreased with age (Supplementary information 1). The odds of being unaware were also higher for participants living at an altitude above 3000 MASL compared to participants living below 2500 MASL (OR: 1.15, CI: 1.03–1.27). Also, having a family history of HTN (OR: 0.82, CI: 0.67–1.00) reduced the odds of being unaware, while a higher LS (OR: 1.06, CI: 1.00–1.14) increased them; however, these associations were not statistically significant. Governmental aid had no influence on the unawareness of HBP (OR: 1.01, CI: 0.93–1.11).

Lastly, in the uncontrolled HBP model, the odds of having uncontrolled HBP increased with age (OR: 1.03, CI: 1.00–1.01) and decreased in participants with a family history of HTN (OR: 0.32, CI: 0.66–1.03), yet the associations were not statistically significant. The remaining variables, sex, living at high altitude, LS and governmental aid did not influence the prevalence of uncontrolled HBP.

## Discussion

This cross-sectional study analysed baseline HBP data for adults aged 30 years and older participating in the Peruvian ALTO cohort. We observed a prevalence of HBP of 18.9%. Notably, 50.1% of men and 41.1% of women aged 75 or older had a HBP. While women were more likely to exhibit HBP at younger ages, men were more likely to exhibit HBP with advancing age. Of all the adults in whom HBP was measured, 72.2% had never been diagnosed with HTN and, thus, were likely unaware of a condition of HTN. HBP unawareness decreased with age, more strongly in men who, at young age, were more likely to be unaware of their condition than women. In our setting, we also found that 58.4% of the patients with a self-reported physician-diagnosed HTN or intake of antihypertensive medication had an uncontrolled HBP. Only 34.8% of the patients reporting a physician-diagnosed HTN received antihypertensive medication. These findings support the need for age and sex-specific interventions for HTN prevention and control.

The prevalence of HBP of almost 19% in our rural Andean study population was slightly lower than the country average (20.6%) but corroborated rates calculated for the Cajamarca Region from the 2018 DHS (16–18%) [10], though the latter had included the adolescent population from 15 years of age onwards. Additionally, the prevalence of HBP was higher compared to data from other studies conducted in Puno (11% in 2010) and Ayacucho (14.6% in 2018), also located in Andean Peru [8,26]. In line with DHS findings at country-level, we observed age differences in HBP prevalence [10]. Moreover, and in contrast to the Peruvian DHS findings prevalence of HBP was higher in women than men in our study, albeit in an age-dependent manner. The different age range of participants and women’s lifestyle in the Andean region could explain these divergent results. While the DHS, analysed data from 15 years of age onwards, our participants were 30 years or older. Hence, women participants in our study are likely to have had children, given that first pregnancies in Peruvian women are likely to occur before the age of 26 [27]. Parity has been associated with higher rates of obesity, especially in rural women in Peru, and potentially a higher risk of HBP [27]. Furthermore, it has been observed that women in our Andean setting might have reduced levels of physical activity and be more exposed to household air pollution compared to men, thus also increasing their risk of HBP at younger ages [28].

To the best of our knowledge, the frequency of unawareness in the ALTO cohort is among the highest at regional level in Peru (72% vs. 38–67%). As found in the Peruvian DHS and in other countries, men, younger participants and people living above 3000 MASL were less aware of their HBP [3,10,11]. This could be explained by the high involvement in and thus, health awareness of women in maternal and child health activities in our setting [29]. However, when women are not pregnant anymore and children are over 5 years of age (i.e. when child growth control visit stops), the use of healthcare services might decrease. Adults only access care in case of symptomatic diseases and not for regular check-ups, explaining the low rates of self-reported comorbidities. Furthermore, the association between HBP unawareness and high altitude may indicate health service access barriers [30].

We found that 36% of participants receiving antihypertensive medication had controlled HBP. In the population-based local Peruvian cohort (CRONICAS) the proportion of successful treatment (i.e. having controlled HBP) among participants receiving antihypertensive medication was similar (40%) [8]. Despite the high unawareness of HBP in our population, treatment success among those medicated was similar to other places. Furthermore, the counter-intuitive observation that controlling HBP was more frequent among non-medicated individuals than among people using antihypertensives and that advanced stages of HBP were more common among those on antihypertensive treatment, may be because people are more likely to obtain antihypertensive treatment if they have advanced stages of HTN. The observation that participants on other medication had the highest levels of stage 1 and higher BP, can reflect a combination of a higher likelihood of being treated at advanced stages of HTN combined with the other medications not working well in controlling BP. The cross-sectional nature of the study does not allow sequential investigation. The counter-intuitive observation of better controlled BP among the non-medicated may also be the result from mistakenly self-reporting a physician-diagnosed of HTN. A study in India found inconsistencies between self-reported HTN without medication and BP measurements, with variation by sex and age categories [31]. People in our setting rarely have their BP measured and a diagnosis of HTN depends on high BP levels at two separate visits. Some participants may have been assumed to have HTN based on the results of BP measurements taken at only one visit to a health centre.

Various factors influence the treatment success of HBP, some of them being an assertive diagnosis, good adherence to treatment, including lifestyle modification and drug use, and continuity of care [32]. The higher levels of poorly controlled BP in patients on antihypertensive medication is likely reflecting that antihypertensive medication in this setting is primarily prescribed for higher stages of HTN, albeit we cannot confirm this hypothesis in our cross-sectional study. Moreover, less than 5% of our participants received a combined treatment (i.e. combination of at least two antihypertensive medications) which can be assigned during follow-ups at a primary or higher-level health establishment when a blood pressure target has not been met [24]. In April 2020, the Peruvian health system experienced a shortage of medicines due to the SARS-CoV-2 pandemic. At the end of 2020, neither the second nor the tertiary health care level reached pre-pandemic dosage numbers of dispensed hypertensives [33]. However, our data cannot distinguish whether the uncontrolled cases observed are due to ineffective treatment, lack of adherence or shortage of medicines.

An important challenge of medication adherence is the asymptomatic characteristic of HBP. People in our setting had identified agitation, pain, weakness, and intense beating of the heart as symptoms of heart diseases [29], though other silent symptoms of HBP such as early morning headaches, nosebleeds, vision changes and buzzing in the ear could be easily ignored. Disease awareness of cardiovascular diseases is lacking in this area [29]. As demonstrated in other settings [29,32] many patients cease medications once symptoms subside. Furthermore, the World Health Organization states that adherence to treatment can be optimised if a combined treatment is given in a single pill, but the Peruvian national guidelines do not specify if this recommendation is followed [24,34]. Age and sex-specific awareness campaigns and regular follow-up care are needed for patients to get acquainted with the side effects due to medication non-adherence and beneficial trade-offs in long-term use of medicines [35]. Strategies to improve treatment adherence, such as tele-monitoring and check-ups performed by non-health professionals (i.e. community health workers) should be evaluated [36,37].

Lastly, consistent with the literature, the odds of HBP increased with a family history of HTN [38] and decreased with improved lifestyle. Our population exhibits low rates of smoking and alcohol consumption, and the population is highly active due to their (subsistence) farming lifestyle. However, villagers have low fruit and vegetable intake, and more than half of the study participants were overweight or obese. Dietary change is a complex factor as it is influenced by individuals’ behaviours and country policies [39,40]. The introduction of front-of-package warning labels for unhealthy foodstuffs was a major change in nutrition policy in Peru in 2013. However, implementation did not occur until 2019 [41].

## Limitations and strengths

This study was carried out based on population-based sampling of pregnancies in a rural province of the Peruvian Andes. International standardised tools were used to collect blood pressure and anthropometric data. However, the study has limitations. First, as the study built on pregnancies as index cases, the results are not generalizable to the whole population. Second, the central idea of the cohort is to study the Andean population; however, people living in our study area do not have ‘Quechua’ as mother tongue and descend from other indigenous groups, compared to central or southern Andean regions. Thus, our study represents the population of northern Andean Peru. Third, causal inference is limited due to the study’s cross-sectional nature, which in particular complicates the interpretation of results related to the effect of antihypertensive treatment on lowering BP and controlling it. Fourth, the self-report of a physician-diagnosed HTN could not be verified nor could the self-reporting on medication use. The cohort was not designed to address HBP unawareness and HBP control, thus, information on continuity of care, drug use and reason for non-adherence to treatment is lacking. Fifth, the study may have been underpowered to identify some determinants of HBP prevalence, underdiagnosis and control. Sample size calculations for the ALTO cohort did not consider the HBP outcomes of this nested cross-sectional study. The sample size for the ALTO cohort was pre-set.

This project could only be realised because it was embedded within a population-based cohort reaching remote communities. Our findings are important for Peru National Public Health, as 30% of its population lives in the Andean region. Furthermore, HBP research requires follow-up of subjects over time, hence this study sets the groundwork for future research on non-communicable diseases in northern Andean Peru.

## Conclusions

We found that 18% of our population had HBP. The prevalence of HBP increased with age, and men over 64 years of age were at higher risk than women. HBP prevalence also increased with family history of HTN and with an unhealthy lifestyle. About 72% of the participants were unaware of their HBP. Unawareness decreased with age, and was higher among men and people living at altitudes above 3000 MASL.

These results emphasise the need for better diagnosis, prevention and control programmes of HBP especially in remote communities designed to specific age and sex groups. Population-based cohorts in rural settings help to detect cases of HBP among people who regularly do not access care. Eventually, they can be used to test effective interventions at times when the Peruvian government is still in the process of implementing the new prevention and control strategies to fight cardiovascular diseases.

## Supplementary Material

Supplemental Material

## Data Availability

The data that support the findings of this study are available from the corresponding author upon request.

## Paper context

Peru shows significant heterogeneity in the prevalence and prevention of high blood pressure across its regions. Understanding the local trends in awareness, treatment, and control of high blood pressure is essential for tailoring disease management to the local context. We found a strong sex-age association with prevalence and unawareness of high blood pressure in the Peruvian Andes. Implementing age and sex-specific interventions is crucial for the effective management of high blood pressure.
