# Supplementary material for: Prevalence, awareness, treatment and control of high blood pressure in a cohort in Northern Andean Peru
Source: Glob Health Action. 2023 Dec 1;16(1):2285100. doi: 10.1080/16549716.2023.2285100 (PMC10795589; doi:10.1080/16549716.2023.2285100)

Supplementary information 1

Title: Prevalence, awareness, treatment and control of high blood pressure in a prospective cohort in northern Andean Peru

Authors: Giuliana Sanchez-Samaniego[1,2] ; Stella Maria Hartinger[1,2,3] ; Daniel Mäusezahl[1,2,] ; Jan Hattendorf[1,2,] ; Günther Fink[1,2]; Nicole Probst-Hensch[1,2]

1. Department of Epidemiology and Public Health, Swiss Tropical and Public Health Institute, Swiss TPH, Allschwil, Switzerland

2. University of Basel, Basel, Switzerland

3. School of Public Health and Administration, Universidad Peruana Cayetano Heredia, UPCH, Lima, Peru

Figure 1 Marginal effects of sex and age interaction in the high blood pressure and unawareness of high blood pressure models.

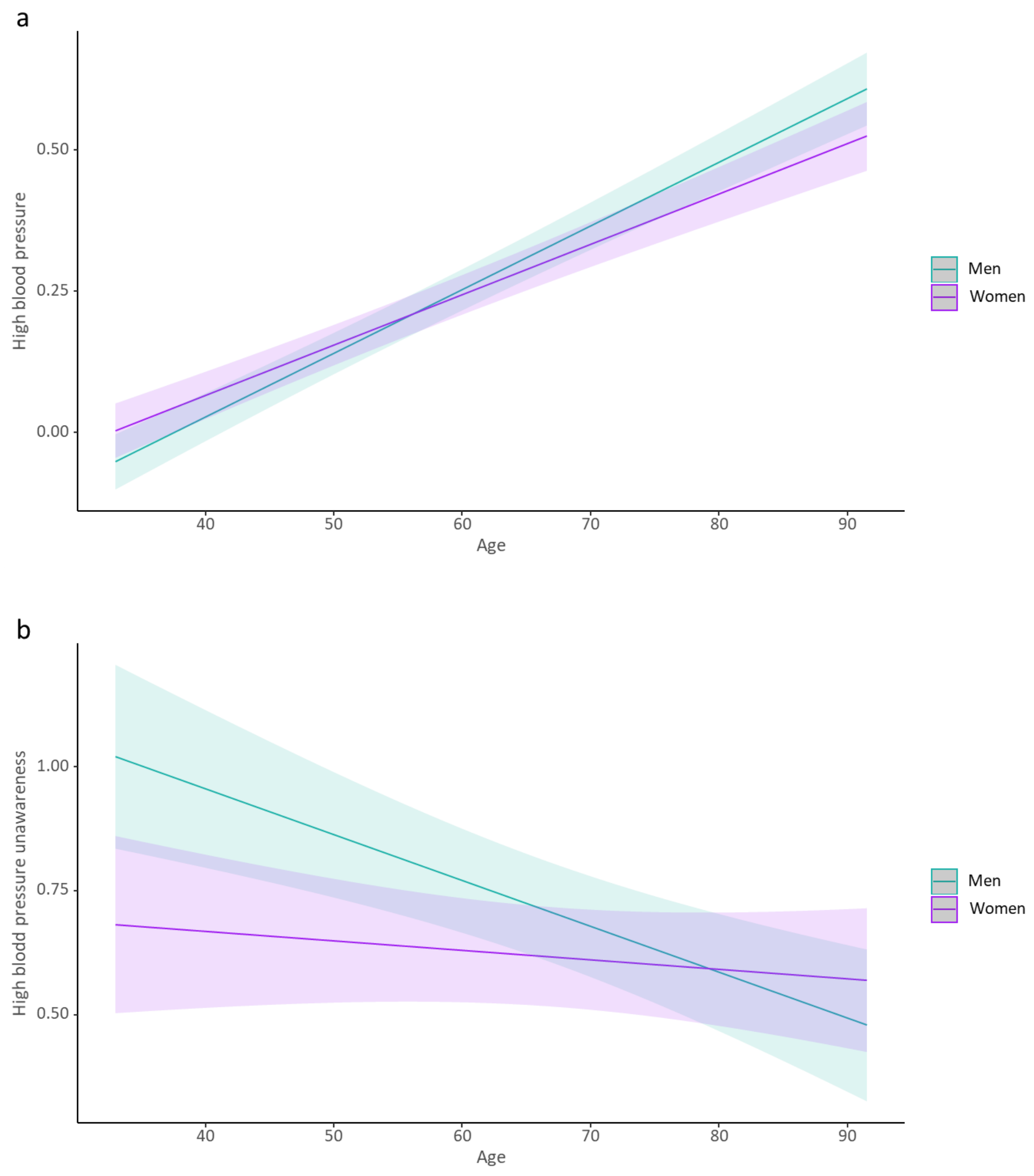

Supplement: Supplemental Material [file ZGHA_A_2285100_SM7392.pdf]
